# Structure and Function of the Bacterial Heterodimeric ABC Transporter CydDC: STIMULATION OF ATPASE ACTIVITY BY THIOL AND HEME COMPOUNDS*

**DOI:** 10.1074/jbc.M114.590414

**Published:** 2014-06-23

**Authors:** Masao Yamashita, Mark Shepherd, Wesley I. Booth, Hao Xie, Vincent Postis, Yvonne Nyathi, Svetomir B. Tzokov, Robert K. Poole, Stephen A. Baldwin, Per A. Bullough

**Affiliations:** From the ‡ Krebs Institute for Biomolecular Research, Department of Molecular Biology and Biotechnology, The University of Sheffield, Sheffield S10 2TN, United Kingdom and; the § School of Biomedical Sciences, The Astbury Centre for Structural Molecular Biology, University of Leeds, Leeds LS2 9JT, United Kingdom

**Keywords:** ABC Transporter, Bacterial Metabolism, Membrane Protein, Membrane Transporter Reconstitution, Microbiology, Protein Structure, Structural Biology, Transporter

## Abstract

In *Escherichia coli*, the biogenesis of both cytochrome *bd*-type quinol oxidases and periplasmic cytochromes requires the ATP-binding cassette-type cysteine/GSH transporter, CydDC. Recombinant CydDC was purified as a heterodimer and found to be an active ATPase both in soluble form with detergent and when reconstituted into a lipid environment. Two-dimensional crystals of CydDC were analyzed by electron cryomicroscopy, and the protein was shown to be made up of two non-identical domains corresponding to the putative CydD and CydC subunits, with dimensions characteristic of other ATP-binding cassette transporters. CydDC binds heme *b.* Detergent-solubilized CydDC appears to adopt at least two structural states, each associated with a characteristic level of bound heme. The purified protein in detergent showed a weak basal ATPase activity (approximately 100 nmol P_i_/min/mg) that was stimulated ∼3-fold by various thiol compounds, suggesting that CydDC could act as a thiol transporter. The presence of heme (either intrinsic or added in the form of hemin) led to a further enhancement of thiol-stimulated ATPase activity, although a large excess of heme inhibited activity. Similar responses of the ATPase activity were observed with CydDC reconstituted into *E. coli* lipids. These results suggest that heme may have a regulatory role in CydDC-mediated transmembrane thiol transport.

## Teaser

**Background:** The ABC transporter CydDC, which pumps sulfur compounds, is required for assembly of the bacterial respiratory machinery.

**Results:** ATP hydrolysis by CydCD in response to sulfur compounds is modulated by hemes.

**Conclusion:** Hemes regulate CydDC in pumping sulfur compounds.

**Significance:** This work is a first step in understanding the structure, function, and regulation of a protein vital to the assembly of the respiratory machinery.

## Introduction

*Escherichia coli* has a complex branched respiratory electron transport system that enables the bacterium to exploit various terminal electron acceptors (1). During aerobic growth, the quinone pool of the inner membrane is used to reduce molecular oxygen to water, the process being mediated by one of the two major membrane-bound respiratory terminal oxidases, cytochrome *bo*′ (encoded by the *cyoABCDE* operon) and *bd*-I (encoded by the *cydAB* operon) (2, 3). Both complexes are electrogenic proton translocators, serving as energy coupling sites for oxidative phosphorylation (4, 5).

Assembly of functional cytochrome *bd*-type quinol oxidases requires not only the structural genes but also the gene products of an unlinked *cydDC* operon (6, 7). On the basis of both the observed gene structure and deduced protein sequences, CydDC is postulated to form a heterodimeric ATP-binding cassette (ABC)^6^ transporter with two transmembrane domains (TMDs), each predicted to comprise six TM α-helices and two nucleotide binding domains (8). The two constituent polypeptides exhibit 27% identity, and the calculated molecular masses for CydD and CydC are 64 and 63 kDa, respectively. Paralogues of CydDC include MsbA (26% sequence identity), an inner membrane lipid flippase that, in turn, is closely related to eukaryotic multidrug export proteins such as P-glycoprotein, MRP1, and MRP2 (9, 10).

The periplasm of *E. coli* mutants lacking functional CydDC has been reported to be “overoxidized” (11). This finding subsequently led to the identification of two substrates for this transporter, cysteine (12) and reduced GSH (13). The phenotype of the *cydD* mutant strain is pleiotropic, exhibiting sensitivity to benzylpenicillin and DTT, loss of motility, and the absence of holocytochrome *c*. These characteristics are also shared by *E. coli dsb* mutants (14), suggesting defective disulfide bond formation/isomerization as a possible cause of these traits in strains lacking a functional CydDC complex. These phenotypes, together with the loss of periplasmic cytochrome *b*_562_ (a typical *cydD* phenotype), can all be corrected by the addition of cysteine or GSH to the growth medium (12, 13). Furthermore, the *cydD* mutant has been shown to contain elevated cysteine levels in the cytoplasm (12). These studies suggest a role for CydDC in relaying the cytoplasmic reducing power to the periplasm.

The dependence of cytochrome *bd* assembly on CydDC also suggests a specific involvement of CydDC in heme processing (15), although a role in heme translocation seems unlikely. Heme incorporation into various exogenous hemoproteins occurs in the periplasm of a *cydC* strain (11), and the uptake of radiolabeled heme into inverted *E. coli* membrane vesicles occurs through a mechanism independent of ATP hydrolysis (15). However, the involvement of CydDC-mediated glutathione/cysteine translocation in hemoprotein assembly is supported by the observation that CydDC overexpression under anaerobic conditions leads to the accumulation of the heme compound P-574 associated with the inner membrane (16). Additionally, anaerobically grown *E. coli*-overexpressing CydDC was subsequently found to contain a heme-bound form of NikA, a periplasmic nickel chaperone (17). These observations point toward a role for CydDC in maintaining an optimum periplasmic redox poise that is required for the incorporation of heme cofactors into respiratory complexes and periplasmic hemoproteins.

To understand more precisely the biochemical role of CydDC, we first need to characterize the nature of the putative heterodimeric assembly and how potential substrates might interact with it. As part of this approach, we attempted to purify the protein for structural studies. Crucial components of these structural studies have been the need to assay ATPase activity and the search for potential substrates and substrate analogues to aid in crystallization. In this work, we provide a direct demonstration of the hitherto putative heterodimeric assembly of CydDC and show, with extensive analysis of a CydDC-heme complex, that the ATPase activity of this ABC transporter responds strongly to thiol and heme compounds.

## EXPERIMENTAL PROCEDURES

### 

#### 

##### Materials

Reagents and their sources were as follows: yeast extract (Oxoid, Cambridge, UK); Bacto-tryptone and bacto-agar (BD Biosciences); *n*-dodecyl-β-d-maltoside (DDM) and *n*-decyl-β-d-maltoside (Glycon, Germany); *E. coli* total/polar lipid extracts (Avanti Polar Lipids, Alabaster, AL); tris(2-carboxyethyl)phosphine (Pierce); phosphotungstic acid (Sigma-Aldrich, Poole, UK); and uranyl formate (Polysciences, Warrington, PA).

##### Cloning and Overexpression of C-His_6_-CydDC

The *cydDC* operon from pRKP1602 (16) was subcloned into the EcoRI and PstI sites of the expression plasmid pTTQ18 (18). The resultant construct, pHX100, encoded CydD with an NS insertion between residues 2 and 3 of the wild-type sequence, arising from the cloning procedure, and CydC bearing a C-terminal SAGGRGSH_6_ tag (C-His_6_-CydDC). The ribosome binding site for translation of the CydD coding sequence is provided by the vector, and a likely ribosome binding site for translation of CydC lies close to the 3′ end of the CydD gene.

*E. coli* BL21 harboring pHX100 was grown in 500 ml of M9 medium containing 50 μg/ml carbenicillin supplemented with 0.2 mm CaCl_2_, 0.2% glycerol, 2 mm MgCl_2_, and 0.2% casamino acid and incubated with shaking at 220 rpm at 37 °C for 16 h in 2-liter conical flasks. Isopropyl 1-thio-β-d-galactopyranoside was then added to a final concentration of 0.6 mm, and the cells were induced for 3 h at 220 rpm and 37 °C. Harvested cells were stored at −70 °C until required.

##### Absorption Spectroscopy

For the whole-cell carbon monoxide difference spectra, *E. coli* AN2342 (*cydD*^+^, referred to as the wild type) and its isogenic *cydD* mutant derivative, AN2343, with/without pHX100, were grown under identical conditions as above. Cells from 500-ml cultures were harvested (2000 × *g*, 20 min, 4 °C) and resuspended in 15 ml of 100 mm phosphate buffer (pH 7.0). The CO-reduced minus reduced difference spectra of whole cells were recorded at room temperature as described in Ref. 19. The protein sample was treated in the same way as described for whole cells, and the identity of bound heme *b* was confirmed by recording pyridine hemochrome difference spectra as in Ref. 20. The Markwell method (21) was used to determine protein concentrations. Heme *b* concentrations were determined using an absorption coefficient of 20.7 mm/cm from ΔA_557–541_ (22).

##### Preparation of E. coli Membrane Fractions

All steps were carried out at 4 °C. Cell pellets were resuspended at 0.4 g (wet weight)/ml in 20 mm Tris-HCl (pH 7.5), 0.5 mm EDTA. After incubation for 30 min, DNase and RNase were added to final concentrations of 20 μg/ml and MgSO_4_ to a final concentration of 10 mm. The suspension was stirred for 30 min, and cells were disrupted by passage through a French pressure cell at 20,000 psi. The lysate was cleared by centrifugation at 10,000 × *g* for 60 min. Membranes were collected by centrifugation at 131,000 × *g* for 90 min. The membrane pellet was washed three times with 20 mm Tris-HCl (pH 7.5) by ultracentrifugation for 60 min at 131,000 × *g* between each wash. The final membrane pellet was homogenized in 20 mm Tris-HCl (pH 7.5) at a protein concentration of 30–60 mg/ml determined using a BCA protein assay kit (Pierce).

##### Solubilization and Purification

All steps were carried out at 4 °C. The washed membranes (240–270 mg protein) were solubilized by incubation for 1 h in 20 mm Tris-HCl (pH 8.0), 20% glycerol, 300 mm NaCl, 10 mm imidazole, and 1% DDM to yield a concentration of ∼4 mg/ml. The remaining insoluble material was pelleted by ultracentrifugation at 100,000 × *g* for 1 h. The supernatant was mixed with 1.5 ml of prewashed (in column equilibration buffer, see below) Ni-NTA resin (Qiagen) and incubated overnight with gentle rocking. The incubated resin was packed into a polypropylene column and washed at 2 ml/min with 70 column volumes of the equilibration buffer (20 mm Tris-HCl (pH 8.0), 20% glycerol, 500 mm KCl, 10 mm imidazole, and 0.1% DDM). The bound protein was eluted at 2 ml/min with 7 column volumes of elution buffer containing 20 mm Tris-HCl (pH 8.0), 20% glycerol, 200 mm imidazole, and 0.02% DDM. Peak fractions were pooled and then concentrated on a Viva-Spin concentrator molecular weight cut-off ((MWCO) 100,000, Sartorius), giving a typical yield of 1.5–2 mg of protein. The latter was then loaded onto three Superdex 200 10/300 GL gel filtration columns connected in tandem (GE Healthcare), equilibrated with gel filtration buffer (20 mm Tris-HCl (pH 8.0), 20% glycerol, and 0.02% DDM), and then the separation was performed on an ÄKTA FPLC system (GE Healthcare) at a flow rate of 0.1 ml/min. Each of the FPLC fractions was analyzed using two wavelengths: 280 nm for the protein and 410 nm for the pigment component. The peak fractions were pooled and stored at −80 °C following snap-freezing in liquid N_2_. No loss in ATPase activity was detected following storage for 2 weeks under these conditions, and 80% of activity was retained following storage at 4 °C for the same period. Proteins prepared for the crystallization trials were used immediately after each of the purification runs.

##### N-terminal Amino Acid Sequencing

The N-terminal amino acid sequences of proteins separated by SDS-PAGE and then transferred by electroblotting to PVDF membrane were determined using a PerkinElmer Life Sciences (Applied Biosystems) model 477A automated amino acid sequencer by Dr. J. N. Keen at the University of Leeds Protein Sequencing Facility.

##### Circular Dichroism Spectroscopy

CD spectra of gel filtration peak fractions were recorded on a Jasco J-810 spectropolarimeter using a 2-mm cell at room temperature. Protein samples were prepared at 0.2 mg/ml in 20 mm phosphate buffer (pH 7.5), 20% glycerol, 0.02% DDM, and 0.01% dimethyl sulfoxide, and spectra were collected from 200–260 nm with a 1.0-nm interval.

##### Reconstitution of C-His_6_-CydDC in E. coli Lipids

Reconstitution of the purified C-His_6_-CydDC was performed essentially as described for MsbA lipid flippase (23). Purified C-His_6_-CydDC was mixed with destabilized (by 2 mm DDM) liposomes of *E. coli* polar lipids (EPL) in 50 mm citrate (trisodium) buffer (pH 6.0), and the detergent was removed by successive additions of BioBeads SM-2 (Bio-Rad) over a period of 2 days. The amount of protein was estimated using the BCA assay. The total phospholipid content was determined as described in Ref. 23.

##### ATPase Assay

The ATPase activity of purified C-His_6_-CydDC (in 0.02% DDM) was measured using a method adapted from Ref. 23. Reaction rates were measured at protein concentrations between 10 and 24 μg/ml. The standard ATPase reaction mixture contained 50 mm Tris (pH 7.5 at 37 °C), 10 mm MgCl_2_, 20% glycerol, 0.02% DDM, and 2 mm ATP. Glycerol and DDM were omitted from the reaction mixture for the reactions with the reconstituted C-His_6_-CydDC. Control reactions received either the gel filtration buffer (for the DDM-solubilized C-His_6_-CydDC) or EPL liposomes containing the equimolar amount of total phospholipids (for the reconstituted protein). Quantification of liberated P_i_ was performed by a colorimetric method (24). The assay was linear up to 400 μm P_i_.

##### Two-dimensional Crystallization of C-His_6-_CydDC

Purified protein (0.1 mg) was mixed with *E. coli* total lipids solubilized in 20 mm Tris-HCl (pH 8.0), containing 20% glycerol and 2% *n*-decyl-β-d-maltoside at lipid:protein weight ratios between 0.2 and 0.4. The total volume of each sample was adjusted to 100 μl with the gel filtration buffer to yield a final protein concentration of 1.0 mg/ml. The mixtures were incubated overnight at 4 °C and then dialyzed against various dialysis buffers (Fig. 5) in a home-built continuous-flow dialysis apparatus over 10 days at 20 °C (25, 26).

##### Labeling with Ni-NTA-Nanogold

Purified C-His_6_-CydDC was mixed with 5 nm Ni-NTA Nanogold particles (Nanoprobes, Yaphank, NY) at a molar ratio of 2:1 (final concentrations of 56 and 28 μm, respectively) in 20 mm Tris-HCl (pH 7.6), 20% glycerol, 150 mm NaCl, and 0.02% DDM and incubated briefly at room temperature. The mixture was concentrated in a Viva Spin 4 concentrator (MWCO 100,000) to 0.5 ml and clarified by centrifugation at 16,000 × *g* for 10 min at 4 °C. Unbound gold particles were separated by applying the supernatant onto a Superose 12 10/300 GL column (GE Healthcare) equilibrated with 20 mm Tris-HCl (pH 7.6), 20% glycerol, 300 mm NaCl, 10 mm imidazole, and 0.02% DDM. Separation was performed on an ÄKTA FPLC system at a flow rate of 0.3 ml/min. Labeling of the C-His_6_-CydDC-EPL proteoliposomes was performed as above, but the glycerol and DDM were omitted from the binding buffer, and the free gold label was removed by washing the liposomes three times in 20 mm Tris-HCl (pH 7.6), 300 mm NaCl, and 10 mm imidazole by passing the pellet through a 27-gauge needle several times, followed by centrifugation at 16,000 × *g* for 10 min at 4 °C.

##### Electron Microscopy

Samples were applied to carbon-coated copper grids (400 mesh, Agar Scientific) after glow-discharging in air. Samples were stained with 0.75% (w/v) uranyl formate or 2% (w/v) phosphotungstic acid. For ice embedding, the sample was applied to a Vitrobot system (FEI), blotted, and frozen in liquid ethane. Imaging of stained samples was performed at a magnification of ∼40,000 on a Philips CM100 at 100 kV, incorporating a Gatan MultiScan 794 1K × 1K charge-coupled device camera. For low-dose (<10 e^−^/Å^2^) cryoimaging, micrographs were recorded at a magnification ∼60,000 on a Philips CM200 FEG operating at 200 kV in conjunction with a Gatan MegaScan 795 2K × 2K charge-coupled device camera.

##### Image Processing

Image processing followed procedures described previously (27, 28) using “MRC” and “*2dx*” software (29).

##### Statistical Analysis

Data points in the kinetic data represent the mean ± S.E. calculated using datasets from three independent measurements. Non-linear curve fitting was performed using either SigmaPlot (Systat Software Inc., San Jose, CA) or OriginPro 7 (OriginLab, Northampton, MA).

## RESULTS

### 

#### 

##### Complementation of a cydD Mutant Strain with His-tagged CydDC

**FIGURE 1.**
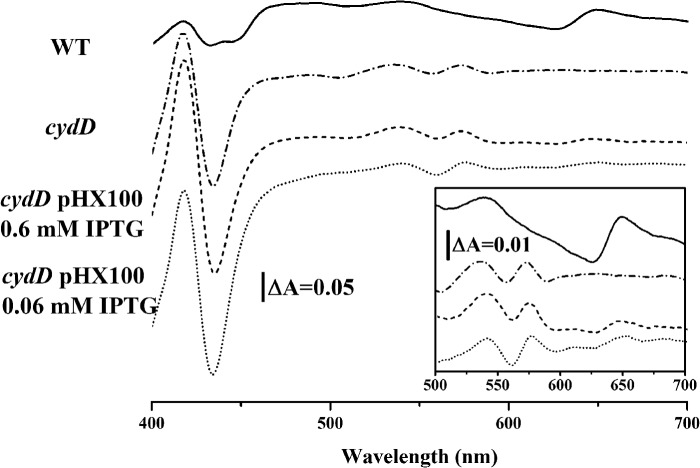
**CO-reduced, minus-reduced, room temperature, whole-cell absorption spectra showing the restoration of the ferrous CO-bound heme *d* signal (maximum around 650 nm, see the *inset* showing the enlarged image of the β- and α-band regions) in the *cydD* mutant *E. coli* strain AN2343 (7) upon transformation with the C-His_6_-CydDC expression plasmid pHX100.** IPTG, isopropyl 1-thio-β-d-galactopyranoside.

Loss of functional CydDC results in the loss of the heme *d* spectroscopic signal associated with the cytochrome *bd* terminal oxidase (7). Fig. 1 confirms that loss of *cydD* abolishes the heme *d* peak at 650 nm and that pHX100 can provide a partial restoration of cytochrome *bd* assembly in *cydD* cells. These data demonstrate that the His-tagged CydDC is functional *in vivo*. The fact that this restoration is partial may be due to the expression of high levels of CydDC being produced by the *tac* promoter. Too much reduced thiol in the periplasm may also perturb heme *d* incorporation.

##### Overexpression and Purification of C-His_6_-CydDC

**FIGURE 2.**
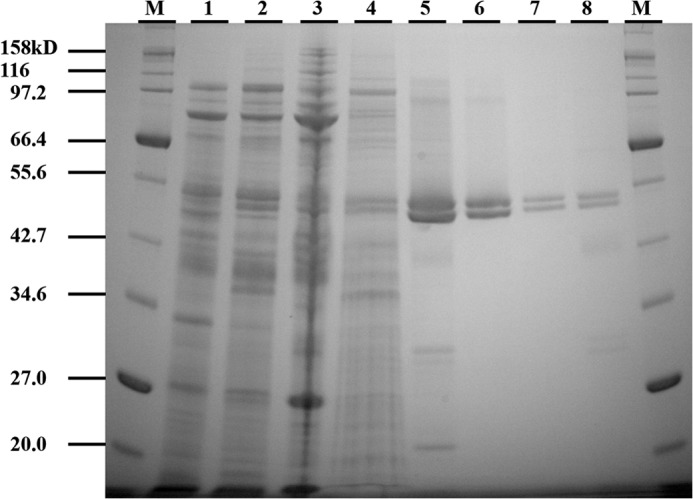
**Coomassie-stained SDS-polyacrylamide gel showing the intermediate samples in the purification.** The amount of protein loaded in each lane was 13 μg unless stated otherwise. *Lane M*, molecular weight markers. *Lane 1*, mixed inner and outer membranes from *E. coli* BL21/pHX100. *Lane 2*, supernatant after incubation with the Ni-NTA resin. *Lane 3*, sedimented pellet (3.3 μg) after solubilization of membranes with 1% DDM. *Lane 4*, proteins eluted from the nickel column during column wash. *Lane 5*, elution from the nickel column with elution buffer (2.0 μg). *Lanes 6* and *7*, elution after gel filtration (as peak 2, see Fig. 3), 2.0 and 0.5 μg, respectively. *Lane 8*, elution after gel filtration (as peak 3, see Fig. 3) (1.0 μg). Electrophoresis was performed using precast NuPAGE^TM^ 10% BisTris gels (Invitrogen) in combination with NuPAGE^TM^ MOPS SDS running buffer (Invitrogen).

**FIGURE 3.**
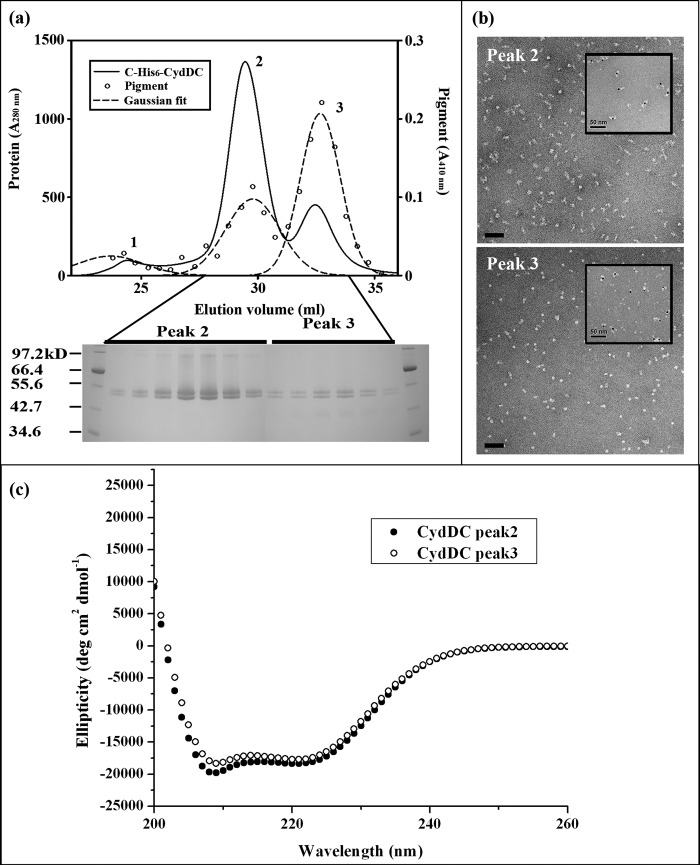
**C-His_6_-CydDC gel filtration chromatography.** *a*, *top panel*, elution profile for the affinity-purified C-His_6_-CydDC from three Superdex200 10/300 GL columns connected in tandem. The *solid line* represents *A*_280 nm_. ○ represent heme absorption (*A*_410 nm_). Peak fitting for the *A*_410 nm_ curve was performed using a three-parameter Gaussian. *Bottom panel*, analysis of the peak fractions by SDS gel electrophoresis. Conditions were as described in the legend for Fig. 2. *b*, electron micrographs showing the C-His_6_-CydDC particles (stained with 2% phosphotungstic acid) for the peak 2 and 3 fractions. *Insets* show equivalent samples labeled with Ni-NTA Nanogold. *c*, CD spectra for the peak 2 and 3 fractions of C-His_6_-CydDC. The spectrum for the CydDC peak 2 overlaps with data points for peak 2 plus 1 μm and 100 nm hemin, respectively.

CydDC was estimated to be expressed at more than 1% of the total inner membrane protein. The SDS gel in Fig. 2 shows that, after a single affinity chromatography step, the preparation is highly enriched in CydDC (Fig. 2, *lane 5*). Subsequent gel filtration chromatography further resolved the affinity-purified protein into three protein peaks (Fig. 3*a*). SDS-PAGE analysis revealed that all of these peaks are comprised of C-His_6_-CydDC (compare *lanes 6–8* in Fig. 2 with the gel image in Fig. 3*a*; peak 1 data not shown). Peak 1 was coincident with the column void volume and yielded protein aggregates. The folding states of CydDC in the two major peak fractions were found to be very similar, with a large α-helical content (Fig. 3*c*). The small spectral differences for the two fractions may indicate differences in conformation or may reflect uncertainty in concentration estimates through variation in iron content. Electron microscopy of peak 2 and 3 fractions revealed well dispersed particles, with those from peak 2 larger and more elongated (approximately 15 nm) than those from peak 3 (approximately 10 nm in the longest dimension) (Fig. 3*b*). Following incubation with Ni-NTA Nanogold, most of these particles were labeled with one gold cluster (Fig. 3*b*, *insets*), suggesting that each particle contains a single CydC subunit. This, together with the appearance of two bands of equal intensity in the gel images in Figs. 2 and 3 (the identities of which were confirmed by sequencing of the six N-terminal residues), indicates that each particle in the electron micrographs in Fig. 3*b* corresponds to a single CydDC heterodimer. The differences in size and shape implied by gel filtration chromatography (Fig. 3*a*) and electron microscopy (Fig. 3*b*) might reflect differences in conformation rather than different aggregation states. However, there was a tendency for particles to aggregate after gold labeling.

The pure protein had a deep brown color, and spectroscopic analysis indicated that this arose from a *b*-type heme. The greater part of this heme component comigrated with peak 3 (Fig. 3*a*). The more populated peak 2 fractions, when pooled, yielded sufficient protein for crystallization trials. The pigment-rich peak 3 fractions were selected for the identification of the heme type by difference spectroscopy. Both fractions were used in the ATPase assays, but the majority of the characterization was performed on the peak 2 fractions, taking advantage of its much greater yield.

##### Identification of the Pigment Component Copurified with C-His_6_-CydDC

**FIGURE 4.**
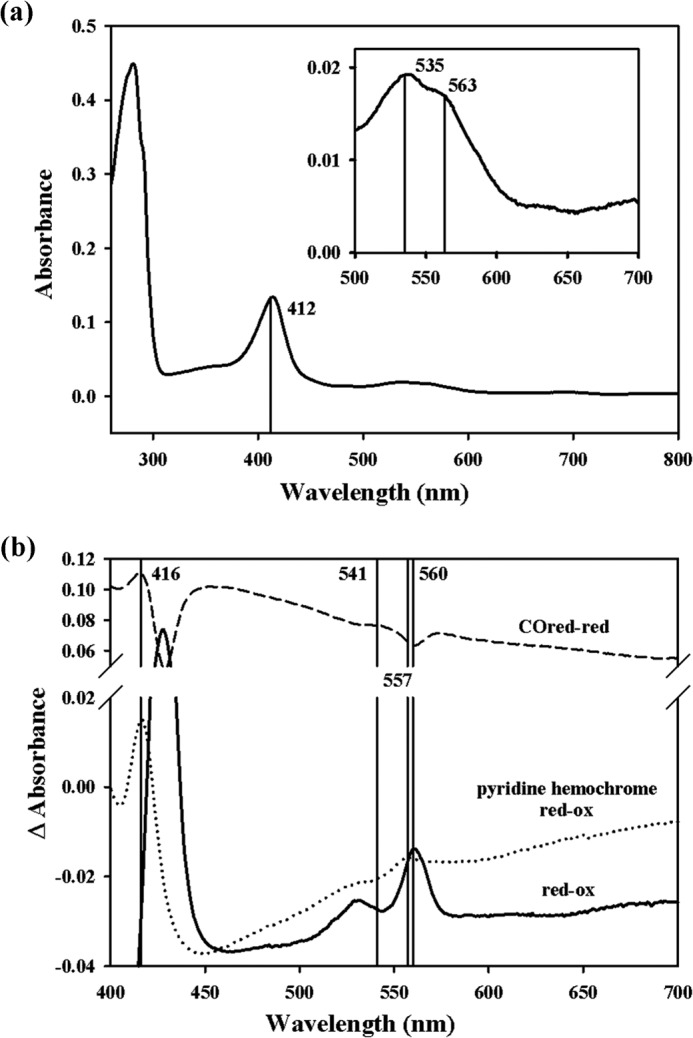
**Identification of heme.** *a*, absorption spectrum of the pooled peak 3 fractions of C-His_6_-CydDC (0.6 mg/ml after concentration), with the *inset* showing the magnified view of the α-band region. *b*, overlay of the three difference spectra (CO-reduced minus reduced (*red-red*), reduced minus oxidized (*red-ox*), and pyridine hemochrome-reduced minus oxidized) with the position of signature peaks indicated. The spectra were offset for clarity.

The absorption spectrum recorded from the pooled peak 3 fractions exhibits a Soret peak at 412 nm and peaks at 535 and 563 nm (Fig. 4*a*). This is characteristic of a low-spin *b*-type heme (30). The reduced minus oxidized pyridine hemochrome difference spectrum displayed peaks at 526 and 556 nm (Fig. 4*b*) that match the peak positions for the pyridine hemochrome of heme *b* (20). The heme *b* concentration in the combined peak 3 fractions (Fig. 3) was measured as 0.87 μm, which gives a heme *b*:protein molar ratio of ∼1:5. Comparison of the peak areas in the *A*_280 nm_ and *A*_410 nm_ traces between peaks 2 and 3 in Fig. 3*a* gives a pigment:protein ratio of ∼1:26 for peak 2.

##### Electron Microscopy of C-His_6_-CydDC Two-dimensional Crystals

**FIGURE 5.**
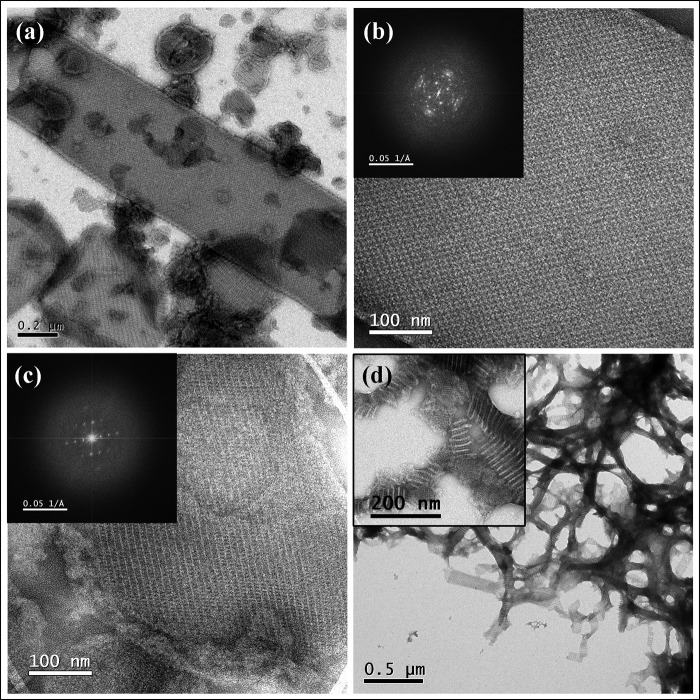
**Gallery of the main forms of two-dimensional crystals of C-His_6_-CydDC.** *a*, low magnification view of the tubular crystal form obtained with 20 mm trisodium citrate (pH 6.0), 20% glycerol, and 0.02% NaN_3_ as the dialysis buffer. *b*, higher magnification view of the tubular crystal form. *Inset*, computer-generated Fourier power spectrum showing spots arising from the two layers of the flattened tube. *c*, platelet crystals obtained with 20 mm *N*-(2-acetamido)-2-iminodiacetic acid (pH 6.0), 20% glycerol, and 0.02% NaN_3_ as the dialysis buffer with the Fourier power spectrum *inset. d*, ribbon-shaped crystals obtained at elevated (*i.e.* >10 mm) Mg^2+^ concentrations in the citrate buffer described in *a. Inset*, enlarged view of the crystals revealing the characteristic striped patterns running perpendicular to the axis of crystal growth. These images were recorded of crystals stained with 0.75% uranyl formate.

**FIGURE 6.**
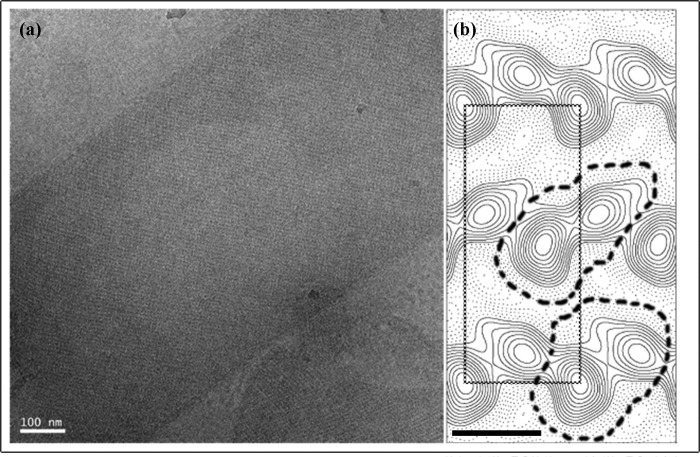
**Image analysis of CydDC crystals.** *a*, low-dose image of the ice-embedded C-His_6_-CydDC tubular crystal recorded at liquid N_2_ temperature. *b*, merged projection map calculated to 30-Å resolution with *p*12_1_ symmetry imposed. One unit cell is outlined, and two possible molecule envelopes are indicated by *thick dashed lines*. Contours are plotted at 0.17 × root-mean-square density, and densities below the mean are represented by *dashed lines. Scale bar* = 50 Å.

**FIGURE 7.**
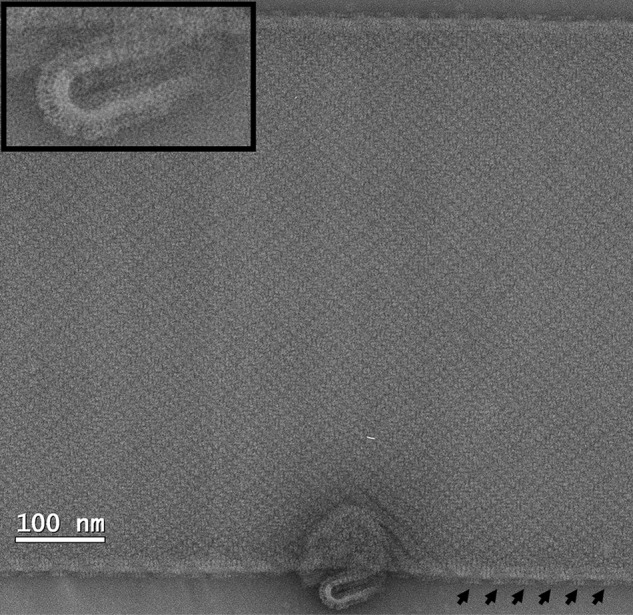
**Low-dose image of a negatively stained tubular crystal of C-His_6_-CydDC.** The *arrows* point out the periodic appearance of the ATPase domains along the longer edge of the crystal, indicating that CydDC is packed in alternating up and down orientations in the crystal. *Inset*, enlarged view of the small vesicle showing the side view along the plane of the lipid layer. The extramembranous ATPase domains are clearly captured in this image. The crystals were stained in 0.75% uranyl formate.

Purified C-His_6_-CydDC was reconstituted into *E. coli* lipids at lipid:protein ratios of 0.2–0.5 (w/w) to form two-dimensional crystals. At the optimum pH of 6.0, CydDC crystallized in three distinct forms, the formations of which were dependent on the type of buffer salt used and also on the concentration of Mg^2+^ in the dialysis buffer (Fig. 5). Tubular crystals were obtained at a particularly high yield. The type of tubular crystal illustrated in Fig. 6*a* proved to be the most tractable for image analysis. Projection maps were calculated from four images of independent crystals, with *a* ∼66 Å, *b* ∼160 Å, and γ ∼90° on average. Unit cell dimensions were highly variable both within and between images, therefore limiting the resolution to about 30 Å. Phases were consistent with *p*12_1_ symmetry. This crystal was formed of an array of dimeric units arranged in alternating “up” and “down” orientations when viewed perpendicular to the putative membrane plane (Fig. 6*b*). Alternating orientations of the extramembrane domain can be seen in “side” view in negatively stained crystals (Fig. 7). The asymmetric unit contains two non-equivalent density peaks that we interpret as representing the densities of the CydD and CydC subunits. Two possible envelopes for the putative CydDC heterodimer are outlined. The projected area enclosed by these two alternative envelopes is consistent with that found for other dimeric ABC transporters (31).

##### ATPase Activity of Purified C-His_6_-CydDC (Peak 2) in 0.02% DDM

**FIGURE 8.**
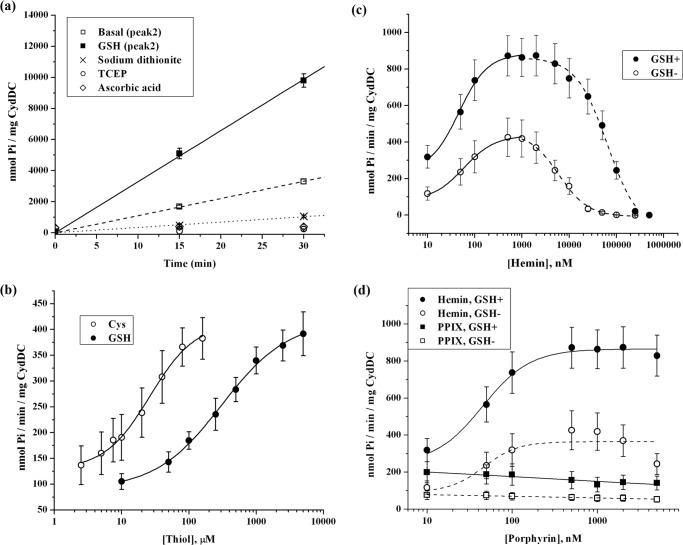
**ATPase activity of purified CydDC (peak 2).** *a*, reaction time courses for the ATPase reaction catalyzed by the peak 2 gel filtration fraction measured in the presence of 5 mm GSH or various non-thiol reductants (fitted lines for the non-thiol reductants (except sodium dithionite) not shown for clarity). Dose-response curves for the ATPase activity of C-His_6_-CydDC (Fig. 3*a*, *peak 2*) in 0.02% DDM with cysteine/GSH (*b*), hemin (in the presence or absence of 5 mm GSH) (*c*), and hemin/PPIX (in the presence or absence of 5 mm GSH) (*d*). The response curves shown for hemin in *d* are derived from the full dose-response curves in *c* to facilitate the comparison with PPIX datasets. Curve fitting through the thiol/hemin datasets in *b*, *c*, and *d* used the logistic dose-response function of the OriginPro 7 software. *c*, the fitting to each response curve dataset was performed separately against two subdatasets, each encompassing data points in the rising (*solid line*) and the falling (*dashed line*) parts of the response curve. Each of the data points represents the mean ± S.E. of multiple (*n* ≥ 3) protein preparations produced from independent membrane preparations. *TCEP*, tris(2-carboxyethyl)phosphine.

**FIGURE 9.**
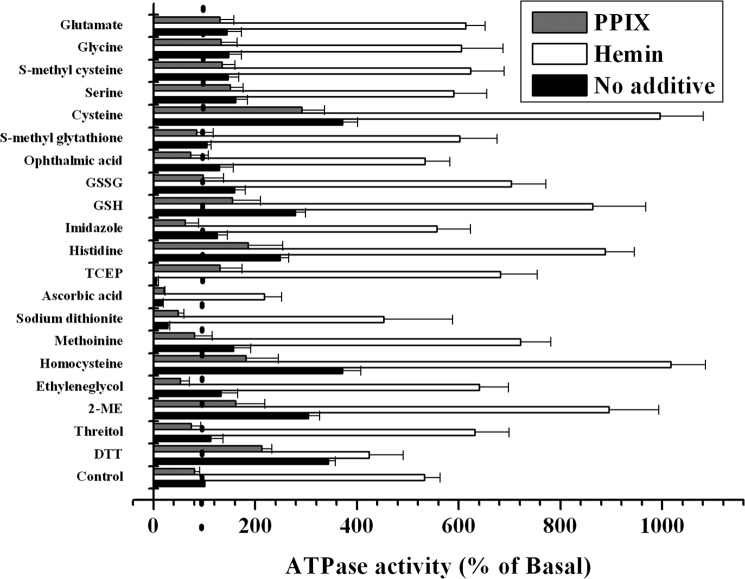
**Modulation of the ATPase activity of purified CydDC in 0.02% DDM by various compounds.** ATPase activity of DDM-solubilized C-His_6_-CydDC (Fig. 3*a*, *peak 2*) was measured in the standard reaction mixture described under “Experimental Procedures.” Test compounds were at 5 mm, except that hemin and protoporphyrin IX were both at 1 μm. Stock solutions of the heme compounds were made either in dimethyl sulfoxide (for hemin) at 20 mm or in 20 mm NaOH (for protoporphyrin IX) at 1 mm. For the reactions with test compounds dissolved in dimethyl sulfoxide or NaOH, parallel control reactions containing these solvents only were performed to correct for their effects on ATPase activity. The *vertical dotted line* at 100% indicates the basal activity level. The basal activity refers to that achieved without any additive(s) and is represented by the *No additive* data in the *Control* category. Each of the data points represents the mean ± S.E. of multiple (*n* ≥ 3) protein preparations produced from independent membrane preparations. *GSSG*, oxidized glutathione (glutathione disulfide); *TCEP*, tris(2-carboxyethyl)phosphine; *2-ME*, 2-mercaptoethanol.

Fig. 8*a* shows that purified C-His_6_-CydDC in 0.02% DDM showed constant Mg^2+^-dependent ATPase activity (approximately 100 nmol P_i_/min/mg) with a linear reaction time course over at least 30 min. This indicated that the structural integrity of the protein was not affected adversely during the time of incubation. Substrate binding to ABC transporters generally leads to the stimulation of their ATPase activities (32). We observed that cysteine and GSH, shown previously to be actively transported by CydDC (12, 13), induced an ∼3- to 4-fold stimulation of the ATPase activity of CydDC (Figs. 8*a* and 9). These thiols stimulated the ATPase activity to similar maximal extents, but cysteine was much more potent (EC_50_ = 26 ± 3 μm) than GSH (EC_50_ = 307 ± 34 μm), as shown in Fig. 8*b*. Other types of thiols, including DTT, 2-mercaptoethanol, and dl-homocysteine, similarly stimulated the ATPase activity of CydDC, as shown in Fig. 9. Inhibition, rather than stimulation, was observed with the three non-thiol reductants: l-ascorbic acid, sodium dithionite, and tris(2-carboxyethyl)phosphine (Fig. 8*a*). This indicates that the thiol-induced stimulation was not merely caused by the reduction of, for example, any of the three cysteine residues of CydDC. The reason for the inhibition of the ATPase activity by the non-thiol reductants is unclear.

We tested the response of the ATPase activity to two types of porphyrin compounds, hemin and protoporphyrin IX (PPIX). As shown in Fig. 8*c*, hemin is a very potent stimulator of CydDC ATPase activity, with the EC_50_ being of the order of 50 nm, but the dose-response curve showed that its effect becomes inhibitory at concentrations above ∼1 μm. At the optimum concentration of 1 μm, hemin alone stimulated the basal ATPase activity 4-fold. Stimulation by hemin was further enhanced by GSH. The basal activity was stimulated synergistically (∼8-fold) by 1 μm hemin plus 5 mm GSH. GSH did not alter the EC_50_ for the hemin-induced stimulation, but it noticeably suppressed the inhibitory effect of hemin. The same dose-response analyses were performed using PPIX, but the ATPase activity did not change in response to this non-iron-containing porphyrin at any concentrations tested (Fig. 8*d*).

Non-thiol structural analogues of the thiols GSH, (homo)cysteine, DTT, and 2-mercaptoethanol (see Fig. 9 for a complete list of the compounds tested) were tested for their ability to stimulate the ATPase activity. Because GSH is a tripeptide comprising glutamate, cysteine, and glycine, the non-thiol amino acid components of GSH were also tested for the stimulation. Histidine and imidazole were tested as additional non-thiol amino compounds. The results are summarized in Fig. 9. Significant changes in ATPase activity were invoked only with thiols and l-histidine, and the extent of the stimulation fell within a similar range of 2- to 3-fold. ATPase activity was also enhanced by many of the other compounds, but the extent of stimulation was much smaller, 1.2- to 1.5-fold.

The response of the ATPase activity of CydDC to the above compounds was next characterized in the presence of the porphyrins hemin or PPIX. As summarized in Fig. 9, a synergistic response to thiols (except DTT) or l-histidine occurred in the presence of the iron protoporphyrin hemin. The extent of the stimulation fell within the range of 8- to 9-fold. Other compounds induced about a 6-fold stimulation in the presence of hemin, but this level of stimulation was only slightly higher than that induced by hemin alone. PPIX did not exhibit any stimulatory activity, either alone or in combination with thiols or l-histidine, and, in many cases, PPIX was seen to slightly inhibit ATPase activity. The reason for the response to tris(2-carboxyethyl)phosphine in the presence of both PPIX and hemin is unclear.

##### ATPase Activity of Purified C-His_6_-CydDC (Peak 3) in 0.02% DDM

**FIGURE 10.**
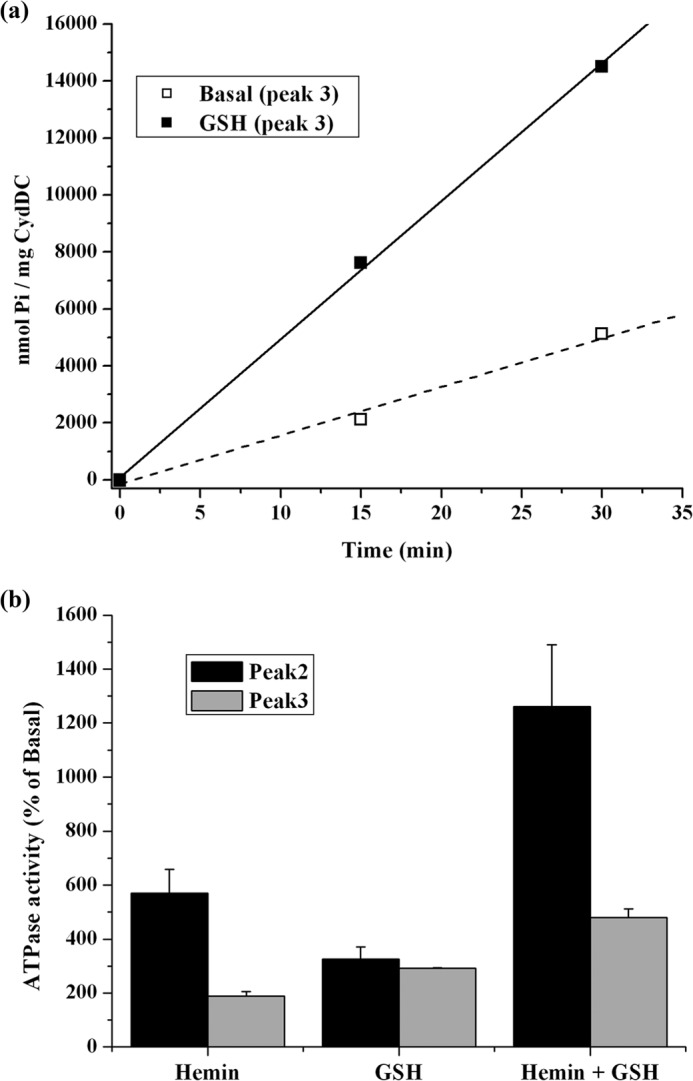
**ATPase activity of purified CydDC (peak 3).** *a*, reaction time courses for the ATPase reaction catalyzed by the peak 3 gel filtration fraction measured in the presence or absence of 5 mm GSH. The activity from this fraction showed an appreciable variability between independent batches. Therefore, a typical result from a single batch is presented. *b*, modulation of the respective basal ATPase activities of the peak 2 and 3 gel filtration fractions by GSH and/or hemin. Each of the data points represents the mean ± S.E. of multiple (*n* ≥ 3) protein batches produced from independent membrane preparations. The activity data shown have been normalized to the basal activities (100%) of the respective peak fractions.

As shown in Fig. 10*a*, the heme *b*-rich CydDC recovered from peak 3 exhibited a linear reaction time course, and the response of its basal activity to GSH was similar to that seen with CydDC in the peak 2 fraction (Fig. 8*a*). However, it showed a basal activity approximately twice as high, and the GSH-stimulated activity was almost comparable with the “synergistically stimulated” activity described for the peak 2 fraction in the presence of added GSH and hemin. By contrast, its response to hemin appeared less marked compared with that of peak 2, as seen in Fig. 10*b*. The response of CydDC in peak 3 to added hemin alone and to simultaneously added hemin and GSH accounted only for 30 and 50% of the extents achieved by CydDC in peak 2, respectively. However, the absolute values for the ATPase activities of the peak 2 and peak 3 fractions in the presence of both saturating hemin and GSH were similar at 863 ± 105 and 660 ± 49 mol P_i_/min/mol CydDC, respectively.

##### ATPase Activity of Purified C-His_6_-CydDC (Peak 2) Reconstituted into E. coli Lipids

**FIGURE 11.**
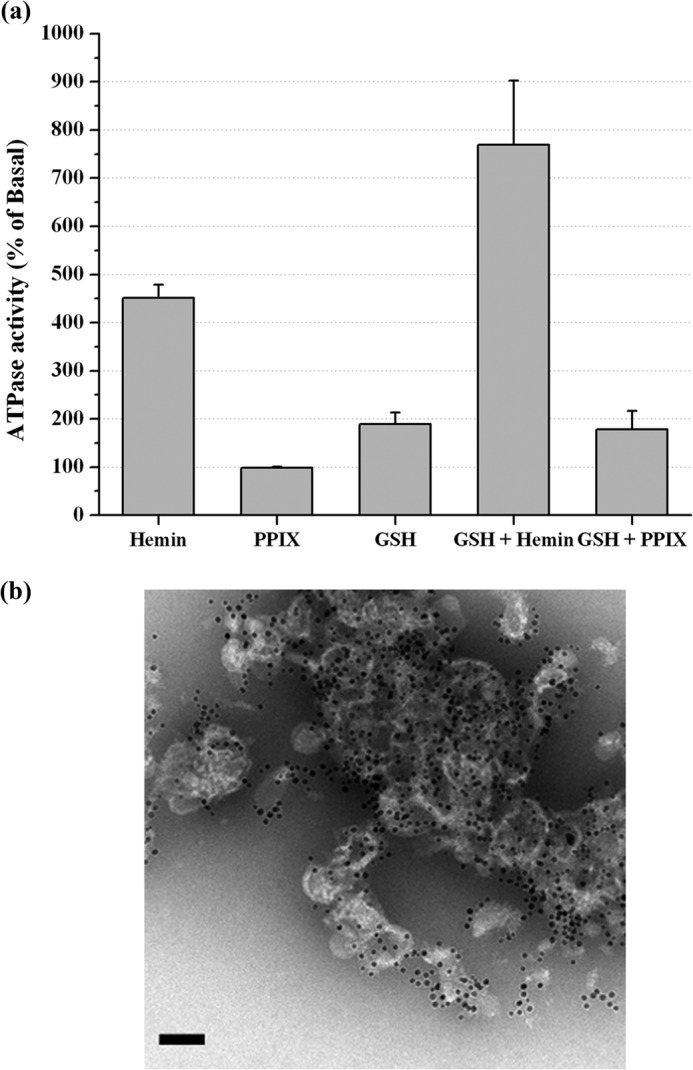
**ATPase activity of CydDC in the presence of lipid.** *a*, ATPase activity of C-His_6_-CydDC (Fig. 3*a*, *peak 2*) reconstituted into *E. coli* lipids. The parallel control runs received equimolar (total phosphorus) amounts of EPL small unilamellar vesicles. The rest of the procedure was the same as for detergent-solubilized C-His_6_-CydDC. Each of the data points represents the mean ± S.E. of multiple (*n* ≥ 3) protein preparations produced from independent membrane batches. *b*, an electron micrograph of the C-His_6_-CydDC-EPL proteoliposomes after labeling with 5.0 nm Ni-NTA Nanogold. The sample was stained in 2% phosphotungstic acid. *Scale bar* = 5 nm.

The effects of a subset of the compounds listed in Fig. 9 on CydDC ATPase activity were tested using C-His_6_-CydDC-EPL proteoliposomes. The result of the reconstitution was checked by electron microscopy after labeling with Ni-NTA Nanogold (Fig. 11*b*). The basal activity from the reconstituted C-His_6_-CydDC showed a greater variability between different preparations than the DDM-solubilized protein, the lowest measured activity being ∼70 nmol P_i_/min/mg, whereas the highest was ∼140 nmol P_i_/min/mg. However, as shown in Fig. 11*a*, the response profile obtained for the reconstituted protein generally correlated well with that of the DDM-solubilized protein. GSH and hemin stimulated the ATPase activity of CydDC 2- and 4-fold, respectively. A strong synergistic 8-fold stimulation of the ATPase activity was induced by the simultaneous presence of GSH and hemin, but PPIX had no stimulatory effect, either alone or in combination with GSH.

## DISCUSSION

### 

#### 

##### Purification of the CydDC Heterodimer in Multiple Conformations

The principal and original aim of this work was to isolate CydDC of sufficient quality and quantity for structure determination. Milligram quantities of monodispersed protein were purified, ATPase activity was demonstrated, and two-dimensional crystallization revealed a clear heterodimeric architecture. Although the genetic evidence is consistent with CydDC functioning as a heterodimeric ABC transporter *in vivo*, this had not been confirmed by any direct means (8, 12, 13). In the overexpressed, His-tagged CydDC, the two subunits are not covalently linked and, therefore, a CydC homodimer could have been an unwanted product. However, the twin and equally staining bands observed by SDS gel electrophoresis (Figs. 2 and 3), the binding of a single gold cluster per protein particle seen in the gold labeling study (Fig. 3*b*, *insets*), and the visualization of two domains of different density within the asymmetric unit of two-dimensional crystals (Fig. 6*b*) provide very strong evidence for the isolation of a heterodimer.

A combination of nickel affinity chromatography and high-resolution gel filtration revealed two distinct populations of protein particles, each associated with a heme *b* component. The most abundant population (Fig. 3*a*, *peak 2*) consisted of slightly elongated particles with dimensions of 6 × 15 nm and a molar heme:protein ratio of 1:26. The minor population (Fig. 3*a*, *peak 3*) consisted of more spherical particles, 6 nm in diameter, with a molar heme:protein ratio of 1:5. When subjected to SDS gel electrophoresis, these two populations appeared identical (Fig. 2). However, the different retention volumes exhibited under gel filtration (Fig. 3*a*), differences in shape revealed by EM (Fig. 3*b*), and slight differences in CD spectra (Fig. 3*c*) all point to the two fractions having different conformations.

##### Crystallization

Although CydDC appears to be a promising candidate for structural studies, the resolution attained currently is sufficient only to reveal domain structure. The tubular type of crystal illustrated in Figs. 5–7 was formed of an array of dimeric units arranged in alternating up and down orientations when viewed perpendicular to the putative membrane plane. It is likely that each unit, consisting of two unequal densities, represents a CydDC heterodimer. The poor crystal order might reflect high conformational flexibility, similar to that inferred from the x-ray crystal structure of MsbA (33). The two populations of CydDC with varying heme content and different dimensions (Fig. 3*a*, *peaks 2* and *3*) indicate the presence of at least two conformations. In any attempt to improve crystal quality, further biochemical characterization will be essential, and this study represents the first steps in this direction.

##### Heme Binding

Purified CydDC is brown. Spectroscopic analysis showed that this originates from copurifying heme *b* (Fig. 4). That a heme can specifically interact with CydDC is also indicated by the strong response of the ATPase activity to nanomolar concentrations of hemin (Fig. 8, *c* and *d*).

The two putative conformers were associated with different levels of copurifying heme (Fig. 3*a*). However, the addition of hemin to the purified CydDC from peak 2 did not retard the gel filtration retention volume to that of peak 3 (data not shown). Similarly, no evidence was seen in the CD spectra or by EM (Fig. 3, *b* and *c*) for a change in conformation upon addition of hemin to the CydDC in peak 2. The higher heme:protein ratio of peak 3 in Fig. 3*a* indicates that an appreciable population of CydDC in this fraction forms a tight complex with a heme *b*. We attempted a second round of gel filtration of the peak 3 fraction, but we observed no equilibrium dissociation of the heme to generate the peak 2 species. Therefore, the putative difference in the conformation of CydDC in the two fractions may be the result of some other factor than heme binding, with the two conformers having different resultant heme-binding occupancies.

##### ATPase Activity Stimulated by Thiols

The His_6_-tagged CydDC construct used in this study provided a partial restoration of cytochrome *bd* assembly in *cydD* cells, demonstrating that the His-tagged CydDC is functional *in vivo*. The partial restoration of the heme *d* signal is not surprising because Poole *et al.* (8) reported that the introduction of the wild-type *cydC*/*D* gene(s) in *trans* to *E. coli cydC*/*D* mutants also resulted in lower levels of heme *d* signal. Nevertheless, the growth of the mutant on selective media was restored.

We focused here on measuring ATPase activity rather than direct transport activity. This information could help in identifying crystallization additives that lock the transporter into one conformation and give insights into CydDC function. Prior to this study, cysteine and GSH were the only confirmed substrates of CydDC (12, 13). Our more extensive survey suggests that CydDC might also mediate the export of other compounds. This could be analogous to members of the ABCC subfamily of ABC transporters, postulated to share a common basic structural architecture with CydDC and including MRP1 and 2.^7^ They have been shown to export both GSH and its conjugates with various physiological and xenobiotic compounds (9). We tested various thiols and their analogues for the stimulation of ATPase activity of the purified transporter in detergent solution (Fig. 9). As reported for P-glycoprotein and other ABC-type import/export systems, nucleotide hydrolysis in the cytosolic nucleotide binding domains is generally stimulated by the binding of the transport substrate in the binding pocket in the TMDs (32). Conversely, those that compete with canonical substrates for the binding site(s) but are lacking the structural determinants for inducing the conformational changes in the TMDs, such as substrate analogues, would not trigger the stimulation of the ATPase activity (23, 35, 36). As summarized in Fig. 9, the ATPase activity of DDM-solubilized CydDC was stimulated by various thiol compounds but not by their *S*-substituted/non-thiol analogues nor by non-thiol reductants. Indeed, the basal activity of CydDC was inhibited markedly by the non-thiol reductants tested (Figs. 8*a* and 9). Our results also revealed a significant response of the CydDC ATPase to l-histidine, but whether or not histidine is involved in protein-heme interaction, as reported for an *E. coli* heme channel, CcsBA (37), awaits further study.

This stimulation profile of CydDC ATPase activity was notably different from that of the purified mammalian ABC-type GSH/GS-X exporter MRP1 (38), where the basal ATPase activity measured in 1% DDM/0.4% sheep brain phospholipids gave 150 nmol/min/mg with an ATP concentration of 2 mm. The ATPase activity was stimulated 1.2-fold by GSH, whereas a somewhat higher stimulation (*i.e.* 1.5-fold) was achieved by oxidized glutathione. CydDC, although showing similar basal activity, showed a greater (3-fold) response to GSH (Fig. 8*a*). Fig. 9 reveals a clear propensity of the ATPase activity of CydDC to respond to thiols. The extent of the stimulation became much less marked when the thiol group was modified or substituted. These data, especially the thiol-dependent ATPase kinetics of CydDC, support the notion that CydDC is unlikely to be a general efflux pump for cysteine/GSH conjugates.

##### ATPase Activity Stimulated by Heme Compounds

As shown in Figs. 8, *c* and *d*, and 9, a dose-dependent increase in the ATPase activity of CydDC (peak 2) by hemin was observed, and the highest activity was reached at a heme:protein ratio of 5:1. At a further excess of hemin, the effect became inhibitory, but this was alleviated in the presence of millimolar concentrations of GSH (Fig. 8*d*). The EC_50_ for hemin was ∼100 nm, which lies close to the reported range of <100 nm for the physiological concentration of heme (39).

Hemin and thiols together induced a strong synergistic stimulation (Figs. 8*c*, 9, and 10*b*). However, neither the EC_50_ for hemin nor the heme:protein ratio for the highest response (*i.e.* 5:1) changed when the dose-response analysis was performed in the presence of GSH. The effect of thiol is, therefore, that its presence increases the turnover rate of the ATPase cycle while not affecting the binding of heme to CydDC.

The greater proportion of heme in the peak 3 fraction appeared to be directly correlated with its approximate 2-fold higher basal ATPase activity relative to that of the peak 2 fraction (Figs. 8*a* and 10*a*). The dose-response curve in Fig. 8*c* shows that, for the 2-fold enhancement of activity in the presence of GSH, a heme concentration of 40 nm is needed. Taking into consideration the protein concentration in the ATPase reaction, this corresponds to a heme:protein ratio of 1:5. The ratio of 1:5 was also estimated from the spectroscopic analysis of the peak 3 fraction. The weaker ATPase response by the CydDC in peak 3 to hemin, as shown in Fig. 10*b*, is consistent with the endogenous heme already having provided an increased relative basal activity.

To exclude the possibility that hemin is a transport substrate, a direct transport assay would be required. However, previous studies showed that CydDC actively exports cysteine and GSH (12, 13) but that it does not mediate an active transport of hemin alone (15). The stimulation of ATPase activity of CydDC by hemin, therefore, suggests the following two possibilities. Heme may be cotransported by CydDC with thiols, or heme may bind and enhance the rate of thiol export by CydDC, but the heme itself is not transported. The second possibility seems most likely. Heme *b* appears to form a stable complex with CydDC in peak 3. Indeed, preincubation of the complex with ATP and GSH at 37 °C (*i.e.* under the same condition as the ATPase assay reaction) in advance of gel filtration did not cause the release of the pigment from CydDC (data not shown). Such a tight interaction would appear to be incompatible with the bound heme *b* as a transport substrate of CydDC. However, the bound heme *b* does modulate the responses of CydDC to both hemin and GSH (Fig. 10*b*). The bound pigment acted to enhance both the basal activity and the response of CydDC to GSH, whereas it suppressed the transporter's response to hemin. The absence of stimulation by PPIX indicates that the stimulation of ATP hydrolysis by CydDC involves the recognition of the central coordinating iron atom of an iron porphyrin (Fig. 8*d*).

This profile of heme stimulation suggests that heme compounds may up-regulate thiol transport. Whether the redox states of the bound heme influence the ATPase activity of CydDC, thereby serving as a mechanism to maintain a proper redox poise in the extracytoplasmic space, was not addressed in this study. It is an intriguing question, left to be answered in future efforts. It is notable that the transport activity of ABC exporters can be modulated by a group of non-substrate compounds, called chemosensitizers, that bind to the TMDs rather than at the binding site for the substrates and affect the conformational coupling between the nucleotide binding domains and the TMDs, therefore controlling transport activity (34). Similar observations have been reported for the rod photoreceptor-specific ABC transporter ABCR (36).

## Footnotes

7 Dean, M., http://www.ncbi.nlm.nih.gov/books/NBK3/.

## Footnotes

6 The abbreviations used are:

ABC: ATP-binding cassette
TMD: transmembrane domain
DDM: *n*-dodecyl-β-d-maltoside
MWCO: molecular weight cut-off
Ni-NTA: nickel-nitrilotriacetic acid
EPL: *E. coli* polar lipid
PPIX: protoporphyrin IX.
